# An Examination into the Effects of a Nutraceutical Supplement on Cognition, Stress, Eye Health, and Skin Satisfaction in Adults with Self-Reported Cognitive Complaints: A Randomized, Double-Blind, Placebo-Controlled Trial

**DOI:** 10.3390/nu16111770

**Published:** 2024-06-05

**Authors:** Adrian L. Lopresti, Stephen J. Smith, Melanie L. Riggs, Rebecca A. Major, Tanner G. Gibb, Steven M. Wood, Shelly N. Hester, Helen E. Knaggs

**Affiliations:** 1 Clinical Research Australia, Perth, WA 6023, Australia; steve@clinicalresearch.com.au; 2 College of Science, Health, Engineering and Education, Murdoch University, Perth, WA 6150, Australia; 3 Pharmanex Research, NSE Products, Inc., Provo, UT 84604, USA

**Keywords:** memory, cognition, nutrients, astaxanthin, vitamin E, grapes, carotenoids

## Abstract

*Background*: Dietary quality and the consumption of antioxidant-rich foods have been shown to protect against memory decline. Therefore, this double-blind, randomized, placebo-controlled study aimed to investigate the effects of a nutritional supplement on changes in cognitive performance. *Methods*: In adults aged 40 to 70 years with subjective memory complaints, participants were randomly allocated to take a supplement containing vitamin E, astaxanthin, and grape juice extract daily for 12 weeks or a matching placebo. The primary outcomes comprised changes in cognitive tasks assessing episodic memory, working memory, and verbal memory. Secondary and exploratory measures included changes in the speed of information processing, attention, and self-report measures of memory, stress, and eye and skin health. Moreover, changes in plasma concentrations of brain-derived neurotrophic factor, malondialdehyde, tumor-necrosis factor-α, and interleukin-6 were measured, along with changes in skin carotenoid concentrations. *Results*: Compared to the placebo, nutritional supplementation was associated with larger improvements in one primary outcome measure comprising episodic memory (*p* = 0.037), but not for working memory (*p* = 0.418) or verbal learning (*p* = 0.841). Findings from secondary and exploratory outcomes demonstrated that the nutraceutical intake was associated with larger improvements in the Everyday Memory Questionnaire (*p* = 0.022), increased plasma brain-derived neurotrophic factor (*p* = 0.030), decreased plasma malondialdehyde (*p* = 0.040), and increased skin carotenoid concentrations (*p* = 0.006). However, there were no group differences in changes in the remaining outcome measures. *Conclusions*: Twelve weeks of supplementation with a nutritional supplement was associated with improvements in episodic memory and several biological markers associated with cognitive health. Future research will be essential to extend and validate the current findings.

## 1. Introduction

In the general population, subjective memory complaints (SMCs) are associated with a reduced global quality of life, increased risk of depression, and negative impact on daily living activities [1,2]. Older people with SMC are twice as likely to develop dementia compared to individuals without SMC. Approximately 2.3% and 6.6% of older people with SMC will progress to dementia and mild cognitive impairment (MCI) per year, respectively [3]. Therefore, identifying interventions to reduce the prevalence of SMC and the potential progression into worsening memory-related conditions is prudent.

Dietary factors have been shown to protect against MCI and dementia, with antioxidant-rich foods possessing specific merit [4,5]. Vitamin E (found in several plant-based oils, nuts, seeds, fruits, and vegetables) is an antioxidant nutrient that has been found to protect against cognitive decline. Even though the evidence is mixed, several trials demonstrate some neuroprotective effects on the progression of MCI [6]. Grapes, a food high in antioxidants, have also been investigated in several trials as a treatment for cognitive decline with several positive findings [7]. Astaxanthin is a red pigment belonging to a group of chemicals called carotenoids with high antioxidant activity. Although research is still preliminary, some studies show it has neuroprotective effects and may protect against cognitive decline [8]. Because of the high antioxidant activity of these nutrients and research demonstrating their neuroprotective effects when delivered in isolation, their delivery as a multi-nutrient supplement for memory and cognitive function was investigated in this study. Moreover, as these nutrients have anti-inflammatory and antioxidant actions, changes in blood markers of inflammation and oxidative stress comprising tumor necrosis factor-alpha (TNF-α), interleukin-6 (IL-6), and malondialdehyde (MDA) were measured. There is consistent evidence that markers of inflammation are elevated in Alzheimer’s disease and some evidence, albeit inconsistent, of elevated inflammation in MCI [9,10,11]. Chronic elevated inflammation is associated with neurodegeneration, impaired neurogenesis, atherosclerotic processes, and chronic disease [12]. Increased markers of oxidative stress, such as MDA, have also been identified in Alzheimer’s disease and MCI, and can contribute to neurodegeneration [13,14]. BDNF is highly concentrated in the hippocampus, is important in synaptic plasticity, contributes to neurogenesis in the dentate gyrus and plays a pivotal role in age-related memory impairments [15]. In a meta-analysis and systematic review, BDNF concentrations were lower in people with Alzheimer’s disease [16], and based on an observational study, low concentrations were identified in older people with MCI [15]. Elevated levels of inflammatory mediators have also been shown to reduce BDNF expression [17], and there is a negative correlation between MDA and BDNF concentrations [18].

Given the preliminary positive evidence of the effects of vitamin E, astaxanthin, and grapes on cognitive health, this study aimed to examine their effects on cognitive health delivered in combination and investigate their potential mechanisms of action. It was hypothesized that this multi-nutrient combination would have positive effects on cognitive performance, particularly on episodic and working memory. Moreover, given their anti-inflammatory and antioxidant activity, it was hypothesized that their neuroprotective effects may be via these mechanisms and, therefore, associated with reductions in TNF-α, IL-6, and MDA. Their impact on skin and eye health was also examined as an exploratory investigation.

## 2. Materials and Methods

### 2.1. Study Design

**Figure 1:**
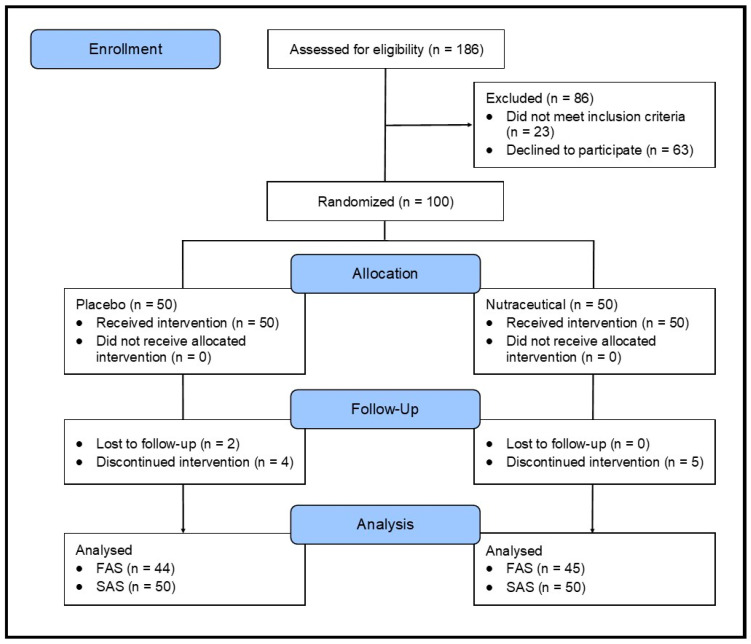
Systematic illustration of study design.

A 12-week, parallel-group, two-arm, single-center, randomized, double-blind, placebo-controlled trial was carried out (Figure 1). The trial protocol was approved by the Human Research Ethics Committee at the National Institute of Integrative Medicine (approval number 0123E_2023), and electronically signed informed consent was obtained from all participants. This study was prospectively registered with ClinicalTrials.gov (ID: NCT05941949).

### 2.2. Sample Size Calculation

An a priori power analysis was conducted to estimate the required sample size. There has been no previous study on the nutraceutical combination investigated in this study, but in previous trials on the cognitive-enhancing effects of herbal ingredients and nutraceuticals, effect sizes of 0.5 to 0.6 have been identified [19,20]. Therefore, an effect size of 0.55 was predicted. Assuming a 5% type 1 error rate (alpha) and a power of 80%, the total number of participants required to find an effect was 84. A 10 to 15% dropout rate was predicted; therefore, it was planned to recruit 100 participants. This was hypothesized to give suitable power to find an effect compared to the placebo, even after dropouts.

### 2.3. Recruitment and Randomization

Volunteers were recruited through social media and e-mail databases between May 2023 and October 2023. Eligible participants were randomly assigned to one of two groups (nutraceutical or placebo; 1:1 ratio). A randomization calculator was utilized to ensure sequence concealment with the randomization structure involving 10 randomly permuted blocks, with 10 participants per block. A participant identification number was allocated based on the order of participant enrollment. The randomization sequence was generated by an investigator not directly involved in recruiting volunteers, and bottle codes were stored by the study sponsor and revealed after all data were analyzed. All softgels were packaged in identical bottles. Study investigators were blind to the treatment allocation until all outcomes were collected and a blind review was completed.

### 2.4. Participants

Interested volunteers, assessed as possibly eligible, were contacted by phone for further assessment of their eligibility and to attain demographic details. The purpose of this 20 min phone interview was to obtain relevant background information, provide volunteers with further information about the study, and evaluate other relevant eligibility criteria. This included obtaining additional information about the participants’ general health, confirmation of medication and supplement use, and previous cognitive assessments. During this telephone interview, the Modified Telephone Interview for Cognitive Status (TICS-M) was also administered. The TICS-M is a 13-item, validated, interviewer-administered screening tool for cognitive impairment and dementia that takes 5 to 7 min to complete [21]. Past research has shown that the TICS-M is as reliable and valid as face-to-face administration and has a specificity of 100% and a sensitivity of 94% in distinguishing healthy controls from individuals with dementia [22].

#### 2.4.1. Inclusion Criteria

The inclusion criteria comprised healthy males and females aged between 40 and 75 years, living in independent accommodation, subjectively reporting memory problems, non-smokers, with a body mass index (BMI) between 18 and 35 kg/m^2^, no plans to start new treatments during the study, able to comprehend the study procedures, and willing and able to adhere with all study procedures.

#### 2.4.2. Exclusion Criteria

The exclusion criteria comprised a diagnosis of dementia based on the revised criteria set by the National Institute on Aging-Alzheimer’s Association (NIA/AA); a score below the 5th percentile for age, education, and sex on the TICS-M; suffering from an unmanaged recently diagnosed medical condition, including but not limited to diabetes, hypertension, endocrine disease, cardiovascular disease, autoimmune disease, gallbladder disease, or cancer/malignancy; a diagnosis of a psychiatric disease (other than mild-to-moderate anxiety or depression); neurological disease such as Parkinson’s or Alzheimer’s disease; a history of seizures, stroke, head injury (with loss of consciousness), or paralysis; regular medication intake, including but not limited to acetylcholinesterase inhibitors, anticholinergics, or steroid medications; a medication change in the last 3 months or an expectation to change during the study period; taking herbal supplements or vitamins that were expected to affect study outcomes; in the previous 6 months, changed the dose or commenced nutritional and/or herbal supplements that may influence treatment outcomes; alcohol intake more than 14 standard drinks per week; a current or 12-month history of illicit drug use; pregnant women; women who were breastfeeding; women who planned to fall pregnant; had a significant surgery over the past year; or a planned significant lifestyle change in the next 3 months.

### 2.5. Intervention

The intervention contained 9 mg astaxanthin, 250 mg grape juice extract, and 12 mg vitamin E (d-alpha tocopherol) daily. This was delivered as 2 softgel capsules once daily with food. The nutraceutical and placebo softgels were identical in appearance, and matched for size, shape, color, and excipients. Excipients in the softgels comprised olive oil, sunflower lecithin, yellow beeswax, fish gelatin, glycerine, and water. Monthly questionnaires assessed adherence to softgel intake, where participants provided an estimate of their intake consistency (0–100%). Moreover, bottles and softgels were returned at the final assessment. Treatment blinding was assessed by requesting participants to guess their group allocation (placebo, nutraceutical, or unsure) at the end of the study.

### 2.6. Outcome Measures

Face-to-face assessments were conducted at baseline (visit 1) and week 12 (visit 2), and self-report questionnaires were completed every 4 weeks (week 0, 4, 8, and 12). During visits 1 and 2, participants completed the Rey Auditory Verbal Learning Test (RAVLT) and several computerized cognitive tasks using the Computerized Mental Performance Assessment System (COMPASS). The COMPASS (Version 6.0) is a software application developed at the Brain, Performance and Nutrition Research Centre at Northumbria University. The cognitive assessments conducted and their order of presentation are described in Supplementary Table S1. All assessments were conducted between 8 and 11 am following an overnight fast. Participants were also instructed not to consume any alcoholic beverage the evening before evaluations and not to consume any caffeinated beverage the morning of each visit.

#### 2.6.1. Primary Outcome Measures

1. Changes in Episodic Memory from baseline to week 12 as measured by the numeric working memory, location learning (delayed recall), and RAVLT delayed recall task (calculations are detailed in Supplementary Table S2).
2. Changes in Working Memory from baseline to week 12 as measured by the corsi blocks and numeric working memory tasks (calculations are detailed in Supplementary Table S2).
3. Changes in Verbal Learning and Memory from baseline to week 12 as measured by the Rey Auditory Verbal Learning Test (RAVLT). The RAVLT is a neuropsychological assessment designed to assess a wide diversity of cognitive functions, including short-term auditory–verbal memory, learning strategies, learning rate, proactive and retroactive interference, information retention, and differences between retrieval and learning [23]. In the RAVLT, the examiner reads out a list of 15 words at one word per second. The participant is then asked to repeat all words from the list that can be remembered. This procedure is repeated five times. The examiner then reads a second list of 15 words (interference list), giving the participant only one attempt to recall this new list. Immediately following this, the participant is asked to recall as many words as possible from the first list. After a 20 min delay, the participant is again asked to remember as many words as possible from the first list. Changes in the total number of words recalled on trials 1 to 5 scores were used to measure verbal learning and memory.

#### 2.6.2. Secondary Outcome Measures

1. Changes in the Everyday Memory Questionnaire—revised (EMQ) total score. The EMQ is a 13-item, reliable and valid self-report measure of memory failure associated with everyday life [24]. Respondents indicate how often they have experienced specific memory problems over the last month.
2. Changes in the Perceived Stress Questionnaire (PSQ) total score. The PSQ is a 30-item self-report questionnaire that assesses a person’s subjective experiences of perceived stressful situations and their stress reactions [25].
3. Changes in the total score for the World Health Organization—5 Wellbeing Index (WHO-5). The WHO-5 is a widely used self-report questionnaire assessing subjective psychological wellbeing [26]. It consists of 5 items that provide a generic global rating of subjective wellbeing.
4. Changes in the Speed of Information Processing from baseline to week 12 as measured by the choice reaction time, simple reaction time, numeric working memory, and digit vigilance task (calculations are detailed in Supplementary Table S2).
5. Changes in the Accuracy of Attention from baseline to week 12 as measured by the choice reaction time task and digit vigilance task (calculations are detailed in Supplementary Table S2).
6. Changes in Visuospatial Learning from baseline to week 12 as measured by displacement scores during the 5 learning trials of the computerized location learning task.
7. A fasting venous blood sample was obtained between 8 and 11 am to measure changes in plasma brain-derived neurotrophic factor (BDNF), malondialdehyde (MDA), Tumor Necrosis Factor-alpha (TNF-α), and Interleukin-6 (IL-6). BDNF plays an important role in learning, memory, and neuronal survival and growth. Disturbances in BDNF have been linked with Alzheimer’s disease and cognitive impairment [27]. MDA is a secondary by-product of cellular lipid peroxidation of polyunsaturated fatty acids and is often used as a biomarker of oxidative stress [28]. During acute inflammation, TNF-α is a cytokine produced by macrophages/monocytes. It is responsible for cell signaling events, leading to necrosis or apoptosis [29]. IL-6 is rapidly and transiently produced in response to infections and tissue injuries and contributes to host defense by stimulating acute phase responses, hematopoiesis, and immune reaction [30].

#### 2.6.3. Exploratory Outcome Measures

1. Changes in the Ocular Surface Disease Index (OSDI) total score. The OSDI assesses ocular irritation symptoms in dry eye disease and how they affect vision-related functions [31]. This 12-item questionnaire assesses dry eye symptoms and their effects on vision-related function in the past week of the patient’s life. A total score ranging from 0 to 100 is calculated. Scores 0 to 12, 13 to 22, 23 to 32 and greater than 33 indicate normal, mild, moderate, and severe dry eye disease, respectively.
2. Changes in Skin Health Satisfaction ratings. On a scale from 0 (none) to 4 (severe), participants rated their skin appearance based on the following criteria: (1) lines, (2) firmness, (3) radiance, (4) texture, (5) hydration, and (6) overall skin appearance.
3. Changes in Skin Carotenoid concentrations were measured using resonance Raman spectroscopy (BioPhotonic Scanner; NSE Products, Provo, UT, USA). In this measurement, blue LED light from the BioPhotonic Scanner was directed on the palm of the hand to measure concentrations of skin carotenoids, measured in Raman Intensity Units (RIUs). Resonance Raman spectroscopy has been shown to accurately measure total carotenoids in human skin with less intra-individual variability than measurements of carotenoids in serum [32]. Skin carotenoids were measured at baseline and week 12.

#### 2.6.4. Safety Measures

To examine the safety and tolerability of softgels, blood assessments of liver and renal function were measured pre- and post-intervention. Changes in blood pressure, BMI, and self-reported adverse events (AEs) (assessed monthly) were also examined over time.

### 2.7. Statistical Analysis

For baseline information, a Pearson’s Chi-square test was used to compare group data for categorical variables and an independent sample *t*-test was used to compare continuous variables. Outcome analyses were conducted on the full analysis set (intention-to-treat). Generalized Linear Mixed Models (GLMMs) were used to assess differences between groups for primary and secondary outcomes over time, with intervention effects assessed through entry of the intervention group (placebo and nutraceutical) × time interaction. The time points for the self-report questionnaires were weeks 0, 4, 8 and 12, and weeks 0 and 12 for the remaining outcome measures. Calculations for the various cognitive measures/domains are detailed in Supplementary Table S2. Random intercepts were used in each model, and covariates sex, age, and BMI were included. Gamma (with log link function) and normal (with identity link function) target distributions were used where applicable. Appropriate covariance structures were used to model correlations associated with repeated time measures in gamma models. Robust estimations were utilized to manage any violations of model assumptions. All data were analyzed using SPSS (version 28; IBM, Armonk, NY, USA) and the *p*-value was set at *p* < 0.05 for all analyses.

## 3. Results

### 3.1. Study Population

A total of 186 people were screened for participation in this study. As specified in Figure 1, 86 people were excluded because they did not meet the eligibility criteria (*n* = 23) or withdrew consent to participate (*n* = 63). The primary reasons for exclusions included withdrawing consent (73.3%), use of prohibited pharmaceutical medications (25.6%), or having a mental health problem (11.6%).

Baseline demographic and clinical characteristics are detailed in Table 1 and Table 2. Analyses revealed that the groups were similarly matched with no statistically significant group differences, except for marital status, where there were more single and less married participants in the placebo group (*p* = 0.027), and there was a greater proportion of participants engaging in high-intensity exercise in the nutraceutical group (*p* = 0.005). An analysis of sex distribution revealed 79% were females and 21% were males, with no differences between the two groups. An assessment of cognitive ability using the TICS-M revealed no group differences in baseline cognitive performance with percentile scores of 44.78% and 49.64% (based on age, sex, and educational level) in the placebo and nutraceutical group, respectively.

### 3.2. Outcome Measures

#### 3.2.1. Primary Outcome Measures

**Figure 2:**
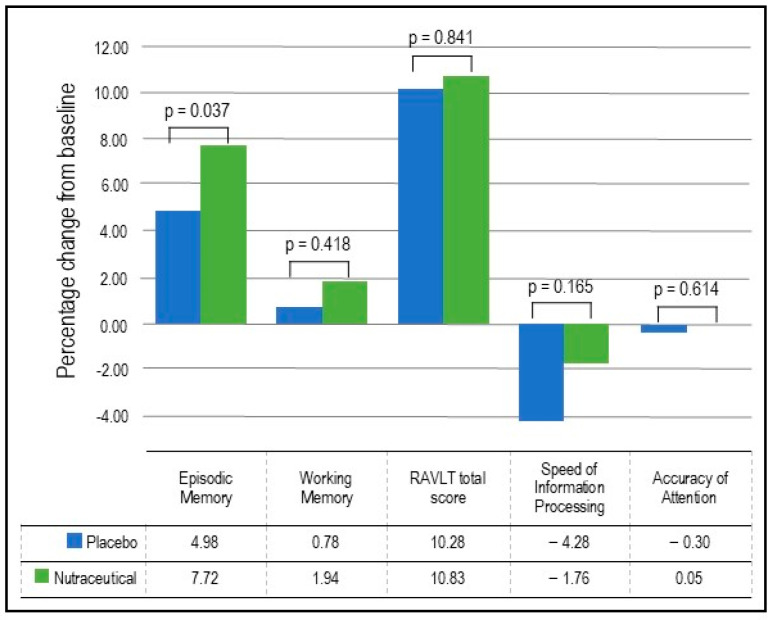
Percentage change in cognitive performance.

1. Episodic Memory: As detailed in Table 3 and Figure 2, the GLMM analysis revealed a statistically significant group × time interaction in episodic memory (*p* = 0.037). In the nutraceutical group, there was a statistically significant 7.72% increase in episodic memory from baseline to week 12 (*p* < 0.001) compared to a statistically significant but smaller 4.98% increase in the placebo group (*p* < 0.001).
2. Working Memory: As detailed in Table 3 and Figure 2, the GLMM analysis revealed no statistically significant group × time interaction in working memory (*p* = 0.418). In the nutraceutical group, there was a near statistically significant 1.94% increase in working memory from baseline to week 12 (*p* = 0.055) compared to a non-significant 0.78% increase in the placebo group (*p* = 0.443).
3. Verbal Learning and Memory: As detailed in Table 3 and Figure 2, the GLMM analysis revealed no statistically significant group × time interaction in the RAVLT total score (*p* = 0.841). In the nutraceutical group, there was a statistically significant 10.83% increase in the RAVLT total score from baseline to week 12 (*p* < 0.001) compared to a 10.28% increase in the placebo group (*p* < 0.001).

#### 3.2.2. Secondary Outcome Measures

**Figure 3:**
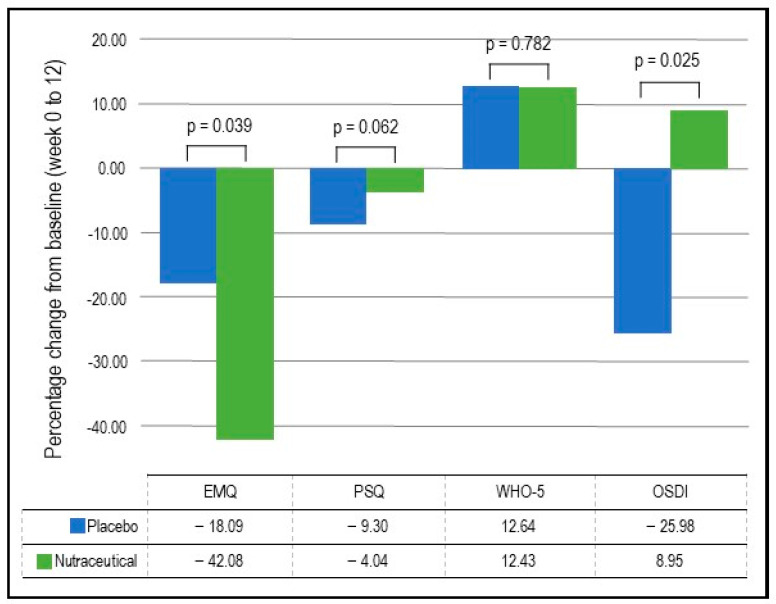
Percentage change in self-report questionnaires.

1. EMQ: As demonstrated in Table 4 and Figure 3, based on the GLMM, there was a statistically significant group × time interaction in EMQ scores (*p* = 0.039). In the nutraceutical group, there was a statistically significant 42.08% decrease in EMQ scores from baseline to week 12 (*p* < 0.001) compared to a statistically significant but smaller 18.09% decrease in the placebo group (*p* = 0.022).
2. PSQ: As demonstrated in Table 4 and Figure 3, based on the GLMM, there was no statistically significant group × time interaction in PSQ scores (*p* = 0.062).
3. WHO-5: As demonstrated in Table 4 and Figure 3, based on the GLMM, there was no statistically significant time × group interaction in WHO-5 scores (*p* = 0.782).
4. Speed of Information Processing: As demonstrated in Table 3 and Figure 2, based on the GLMM, there was no statistically significant group × time interaction in the speed of information processing (*p* = 0.165).
5. Accuracy of Attention: As demonstrated in Table 3 and Figure 2, based on the GLMM, there was no statistically significant group × time interaction in the accuracy of attention (*p* = 0.614).
6. Visuospatial learning (Computerized Location Learning Task): Based on the GLMM, there was no statistically significant group × time interaction in the location learning total displacement score from trials 1 to 5 (*p* = 0.715).
7. Blood markers: As demonstrated in Table 5, based on the GLMM, there were statistically significant time × group interactions for BDNF (*p* = 0.030) and MDA concentrations (*p* = 0.040). However, there were no statistically significant group × time interactions for TNF-α (*p* = 0.445) and IL-6 (*p* = 0.691). In the nutraceutical group, there was a statistically significant 46.10% increase in BDNF concentrations from baseline to week 12 (*p* < 0.001) compared to a 24.41% increase in the placebo group (*p* < 0.001). Regarding changes in MDA concentrations over time, there was a non-significant 12.49% decrease in the nutraceutical group (*p* = 0.294) and a near-significant 26.52% increase in the placebo group (*p* = 0.072).
8. As there was significant variability in the measured blood concentrations, a non-parametric test (independent-sample Mann–Whitney U test) was conducted to confirm if there were any statistically significant group differences in changes in blood concentrations from baseline to week 12. These analyses demonstrated that there were statistically significant group differences in changes in BDNF (*p* = 0.037) and MDA (*p* = 0.020), but not TNF-α (*p* = 0.531) and IL-6 (*p* = 0.728).

#### 3.2.3. Exploratory Outcome Measures

1. OSDI: As detailed in Table 4 and Figure 3, based on the GLMM, there was a statistically significant group × time interaction in OSDI scores (*p* = 0.025). In the placebo group, there was a statistically significant 25.98% decrease (improvement) in OSDI scores from baseline to week 12 (*p* = 0.002) compared to a non-significant 8.95% increase in the nutraceutical group (*p* = 0.297).
2. Skin Carotenoid Concentrations: As detailed in Table 5, the GLMM analysis revealed a statistically significant group × time interaction in skin carotenoid concentrations (*p* = 0.006). In the nutraceutical group, there was a statistically significant 9.16% increase in carotenoid concentrations from baseline to week 12 (*p* = 0.014) compared to a non-significant 5.13% decrease in the placebo group (*p* = 0.143).
3. Skin Health Satisfaction: Based on the GLMM, there was a statistically significant group × time interaction in ratings of facial radiance (*p* = 0.014). In the placebo group, there was a statistically significant 6.48% decrease in self-ratings (improvement) from baseline to week 12 (*p* < 0.001) compared to a non-significant 3.09% increase in the nutraceutical group (*p* = 0.487). There were no statistically significant group × time interactions for other skin satisfaction ratings.
4. Individual Cognitive Tasks: As detailed in Supplementary Table S3, based on the GLMM, there was a statistically significant group × time interaction in the Numeric Working Memory (NWM) task for percentage correct responses (*p* = 0.014) and computerized location learning (CLL) recall displacement score (*p* < 0.001). In the placebo group, there was a non-significant 0.30% increase in correct responses for the NWM task (*p* = 0.755) and a non-significant 23.03% decrease in the displacement score (indicating an improvement) for the CLL recall task (*p* = 0.148). However, in the nutraceutical group, there was a statistically significant 3.65% increase in correct responses for the NWM task (*p* < 0.001) and a statistically significant 75.04% decrease in the displacement score for the CLL recall task (*p* < 0.001). There were no between-group differences in the performance of other cognitive tasks.

#### 3.2.4. Safety and Tolerability

There were no differences between the groups in changes in weight, BMI or blood pressure over time. Participants reported no serious AEs, and the frequency of AEs was similar in both groups. Table 6 details the AEs that were possibly or probably related to the investigational products. There was a higher frequency of mild treatment-related AEs in the nutraceutical group (10%) compared to the placebo group (2%). The most common treatment-related AE in the nutraceutical group comprised gastrointestinal symptoms, which occurred in three participants (6%).

Changes in safety blood markers comprising liver and renal function were examined over time and no participants experienced clinically significant changes in blood markers, with concentrations remaining within or close to established reference ranges. Moreover, changes in blood markers from baseline to week 12 were similar between the placebo and nutraceutical groups, except for a statistically significant between-group difference in changes in bicarbonate concentrations. This was primarily attributable to a decrease in bicarbonate in the placebo group. However, this difference between groups must be viewed tentatively as the number of analyses conducted increased the likelihood of type 1 errors. Moreover, changes in this marker were small and not clinically meaningful.

#### 3.2.5. Treatment Discontinuation

A total of 11 people discontinued the study, including 6 in the placebo group and 5 in the nutraceutical group. In the nutraceutical group, reasons for discontinuation comprised increased family-related stressors (*n* = 1), complications associated with long COVID-19 (*n* = 1), digestive discomfort believed to be associated with softgel intake (*n* = 1), illness unrelated to investigational product (*n* = 1), and a deterioration in mood (*n* = 1). In the placebo group, the reasons for discontinuation comprised the following: no reason given (*n* = 2), moving interstate (*n* = 1), believing study tasks were too onerous (*n* = 1), COVID-19 illness at the time of final visit (*n* = 1), and mood deterioration (*n* = 1).

#### 3.2.6. Effectiveness of Participant Blinding

To assess the efficacy of group concealment during the trial, participants predicted their group allocation (i.e., placebo, nutraceutical, or unsure) at the end of the study. Overall group concealment was high, as 64% of participants were unsure or incorrectly guessed treatment allocation.

## 4. Discussion

In this 12-week, two-arm, parallel-group, randomized, double-blind, placebo-controlled study, the effects of a nutraceutical containing astaxanthin, vitamin E, and grape juice extract on cognitive performance, mood, skin, and eye health were examined in adults with self-reported memory complaints. The primary outcomes of this study comprised an examination of changes in episodic memory, working memory, and verbal learning/memory. Based on the completion of several tasks assessing episodic memory, the nutraceutical was associated with significantly larger improvements in episodic memory compared to the placebo group. However, there were no significant differences between the groups in changes in the other primary outcome measures comprising working memory and verbal learning. Moreover, secondary analyses of changes in other objective measures of cognitive performance comprising accuracy of attention, speed of information processing, and visuospatial learning also demonstrated no significant group differences. However, based on the results from the Everyday Memory Questionnaire, a secondary outcome measure and self-report assessment of cognitive performance, participants in the nutraceutical group reported larger memory improvements over time (42% improvement) compared to the placebo group (18% improvement). Other self-report measures of stress, general wellbeing, and eye and skin health demonstrated no difference between the nutraceutical and placebo groups.

Episodic memory is a cognitive process involving the retention, recall, and encoding of information about experiences and events concerning a specific time and place. During aging, episodic memory declines and has been suggested to be the most age-sensitive memory system [33]. A worsening in episodic memory is one of the earliest and most common symptoms of mild cognitive impairment, and in people with Alzheimer’s disease, severe deficits are present in this cognitive process [34,35]. It is believed that episodic memory is mediated by the circuity of the medial temporal lobe, including the hippocampus, which interacts with other cortical and subcortical structures [36]. Therefore, given the promising improvements in episodic memory identified in this study, it is postulated that the nutraceutical may have protective effects in this brain region. However, this requires confirmation in future trials.

Despite improvements in episodic memory, there were no significant group differences in measures of working memory, verbal learning/memory, speed of information processing, accuracy of attention, and visuospatial learning. These null effects suggest the nutraceutical administered for 12 weeks did not influence these cognitive domains and brain regions important for their function. Many of the administered tasks used to measure these domains required sustained focus, attention, and vigilance, which the nutraceutical seemed to have little impact on. However, it is possible that as participants with deficits in these areas were not specifically recruited in the study, future trials in people with attentional and working memory deficits will be important to substantiate these null findings. Moreover, no group differences in changes in self-reported stress, visual function, and skin satisfaction were identified, but participants with difficulties in these areas were again not explicitly recruited.

Blood and skin measures were assessed over time to help understand the potential mechanisms of action of the nutraceutical. These measures demonstrated that the nutraceutical significantly increased BDNF concentrations compared to the placebo, and there was a statistically significant group difference in changes in MDA concentrations. Moreover, skin carotenoid concentrations, measured by resonance Raman spectroscopy (BioPhotonic Scanner), increased in the nutraceutical group compared to the placebo group. As carotenoids have strong antioxidant effects and MDA is a biomarker of oxidative stress/lipid peroxidation, the increases in skin carotenoid levels and reductions in MDA suggest the nutraceutical may have neuroprotective effects via its antioxidant activity. A meta-analysis of randomized intervention trials reported that carotenoid interventions are associated with better cognitive performance [37]. Moreover, increased oxidative stress is believed to be associated with neurodegeneration and cognitive decline [38]. In a study of patients with recurrent depressive disorder, a higher concentration of plasma MDA was associated with worsened cognitive performance [39]. The increases in plasma BDNF from the nutraceutical intervention also suggest its neuroprotective effects may be via its ability to increase this important neurotrophin. BDNF is essential for neuronal survival and growth, and the signaling cascades initiated by BDNF and its receptors are critical regulators of synaptic plasticity [40].

Overall, the nutraceutical was well-tolerated as there were no reported serious adverse reactions or significant changes in safety blood markers comprising renal and liver function measures. Moreover, there were no changes in weight or blood pressure over time. Participant self-reports indicated that the nutraceutical may be associated with an increased risk of mild, transient gastrointestinal symptoms comprising nausea and bloating. However, this was only reported in three participants (6%) who received the nutraceutical.

## 5. Conclusions

The results from this 12-week study provide some support for the cognitive-enhancing effects of a nutraceutical containing astaxanthin, vitamin E, and grape juice extract in adults with self-reported memory complaints. This was demonstrated by improvements in one primary outcome measure (episodic memory) but not working memory or verbal learning. Cognitive benefits may be achieved via the nutraceutical increasing BDNF (a neurotrophin) and reducing MDA (oxidative stress marker) concentrations. Future studies conducted on larger samples, using more comprehensive cognitive assessments and assessing additional mechanisms of action, will help expand and validate the promising findings from this study. Moreover, although participants with self-reported cognitive complaints were recruited in this study, based on the results of a validated cognitive assessment (TICS-M), the recruited population presented with minimal-to-no cognitive deficits as, overall, they scored within the expected age-appropriate levels at baseline. Therefore, conducting trials on populations presenting with cognitive deficits, such as people with established MCI, will help understand the efficacy of this nutraceutical in preventing further cognitive decline or enhancing cognitive performance over time.

## Figures and Tables

**Table 1:** Baseline clinical characteristics and sociodemographic.

		Placebo (*n* = 50)	Nutraceutical (*n* = 50)	*p*-Value
Age	Mean	58.93	59.82	0.564 ^
	SE	1.18	0.99	
Sex	Female (*n*)	40	39	0.806 ^#^
	Male (*n*)	10	11	
Height (m)	Mean	1.69	1.69	0.690 ^
	SE	0.01	0.01	
Weight (kg)	Mean	77.26	75.92	0.826 ^
	SE	2.54	2.20	
BMI	Mean	26.76	26.48	0.765 ^
	SE	0.69	0.64	
Systolic blood pressure (mmHg)	Mean	130.82	131.00	0.956 ^
	SE	1.93	2.63	
Diastolic blood pressure (mmHg)	Mean	81.26	81.54	0.895 ^
	SE	1.30	1.68	
Marital status	Single	28	17	0.027 ^
	Married/defacto	22	33	
TICS-M percentile (based on age, sex, and education	Mean	44.78	49.64	0.239 ^
	SE	3.03	2.78	
Educational level	Secondary	25	28	0.626 ^#^
	Tertiary	12	13	
	Post-graduate	13	9	
IPAQ category	Low	29	20	0.005 ^#^
	Moderate	21	21	
	High	0	9	
Occupation	Retired	15	11	0.697 ^#^
	Professional	12	10	
	Services and sales worker	6	9	
	Unemployed	5	4	
	Technicians and associate professionals	2	2	
	Elementary occupation	1	3	
	Plant and machine operators and assemblers	0	2	
	Clerical support worker	2	5	
	Craft and related trades worker	1	1	
	Student	1	0	
	Manager	5	3	

^ Independent samples *t*-test; ^#^ Chi-square test.

**Table 2:** Baseline clinical characteristics.

		Placebo (*n* = 50)	Nutraceutical (*n* = 50)	*p*-Value
EMQ	Mean	14.28	16.50	0.376 ^
	SE	1.20	1.40	
PSQ	Mean	64.62	67.90	0.310 ^
	SE	1.96	2.54	
WHO-5	Mean	13.62	12.54	0.311 ^
	SE	0.63	0.85	
OSDI	Mean	18.78	18.42	0.906 ^
	SE	2.29	2.01	
BioPhotonic Scanner score (Raman Intensity Units (RIUs))	Mean	31,040	32,600	0.464 ^
	SE	1451.80	1550.77	
RAVLT—total recalled (trials 1 to 5)	Mean	54.10	51.00	0.085 ^
	SE	1.28	1.24	

^ Independent samples *t*-test.

**Table 3:** Change in cognitive domains (estimated marginal means).

		Placebo (*n* = 44)				Nutraceutical (*n* = 45)				*p*-Value ^b^
		Week 0	Week 12	% Change	*p*-Value ^a^	Week 0	Week 12	% Change	*p*-Value ^a^	
Episodic Memory	Mean	85.87	90.15	4.98	<0.001	85.22	91.80	7.72	<0.001	0.037
	SE	0.48	0.54			0.48	0.54			
Working Memory	Mean	65.10	65.61	0.78	0.443	65.21	66.47	1.94	0.055	0.418
	SE	0.84	0.87			0.83	0.86			
RAVLT—Total Score (Trials 1 to 5)	Mean	53.54	59.05	10.28	<0.001	50.85	56.36	10.83	<0.001	0.841
	SE	1.36	1.54			1.26	1.43			
Speed of Information Processing	Mean	575.66	551.05	−4.28	0.001	574.50	564.40	−1.76	<0.001	0.165
	SE	12.25	11.93			11.92	11.90			
Accuracy of Attention	Mean	95.39	95.10	−0.30	0.545	95.89	95.94	0.05	0.917	0.614
	SE	0.90	0.91			0.88	0.89			

The results (estimated means) are generated from generalized mixed-effects models adjusted for sex, age, and BMI. ^a^ *p*-values are generated from repeated measures generalized mixed-effects models adjusted for sex, age, and BMI (time effects—baseline to week 12). ^b^ *p*-values are generated from repeated measures generalized mixed-effects models adjusted for sex, age, and BMI (time × group interaction).

**Table 4:** Changes in self-report measures (estimated marginal means).

		Placebo (*n* = 44)						Nutraceutical (*n* = 45)						*p*-Value ^b^
		Week 0	Week 4	Week 8	Week 12	% Change	*p*-Value ^a^	Week 0	Week 4	Week 8	Week 12	% Change	*p*-Value ^a^	
EMQ	Mean	12.22	10.22	9.70	10.01	−18.09	0.022	14.59	10.53	10.06	8.45	−42.08	<0.001	0.039
	SE	1.33	1.13	1.08	1.11			1.54	1.13	1.08	0.92			
PSQ	Mean	62.67	61.63	59.28	56.84	−9.30	<0.001	66.66	66.90	67.56	63.97	−4.04	0.044	0.062
	SE	2.33	2.32	2.24	2.15			2.42	2.44	2.48	2.35			
WHO-5	Mean	14.24	14.94	14.75	16.04	12.64	0.01	12.87	12.92	12.68	14.47	12.43	0.011	0.782
	SE	0.87	0.93	0.92	1.01			0.77	0.78	0.77	0.88			
OSDI	Mean	17.01	14.94	13.95	12.59	−25.98	0.002	16.75	15.76	15.91	18.25	8.95	0.297	0.025
	SE	2.37	2.39	2.40	2.41			2.32	2.32	2.34	2.35			

The results (estimated means) are created from generalized mixed-effects models adjusted for age, sex, and BMI. ^a^ *p*-values are produced from repeated measures generalized mixed-effects models adjusted for age, sex, BMI (time effects—baseline and week 12). ^b^ *p*-values are produced from repeated measures generalized mixed-effects models adjusted for age, sex, and BMI (group × time interaction).

**Table 5:** Change in blood markers and skin carotenoids (estimated marginal means).

		Placebo				Nutraceutical				*p*-Value ^b^
		Week 0	Week 12	% Change	*p*-Value ^a^	Week 0	Week 12	% Change	*p*-Value ^a^	
	N	40				43				
BDNF pg/mL	Mean	666.37	829.06	24.41	<0.001	575.45	840.74	46.1	<0.001	0.030
	SE	37.92	46.93			31.38	46.51			
MDA ng/mL	Mean	27.02	34.18	26.52	0.072	28.56	25	−12.49	0.294	0.040
	SE	3.94	4.96			3.99	3.53			
TNF-α pg/mL	Mean	25.99	19.14	−26.37	0.049	20.21	17.09	−15.43	0.217	0.445
	SE	7.4	5.44			5.49	4.66			
IL-6 pg/mL	Mean	32.87	33.59	2.21	0.962	38.33	50.63	32.09	0.55	0.691
	SE	11.05	11.21			12.48	16.82			
Skin carotenoid concentrations (RIU)	N	44				45				
	Mean	29,874	28,341	−5.13	0.143	31,498	34,383	9.16	0.014	0.006
	SE	1644	1590			1689	1872			

The results (estimated means) are created from generalized mixed-effects models adjusted for age, sex, and BMI. ^a^ *p*-values are produced from repeated measures generalized mixed-effects models adjusted for age, sex, BMI (time effects—baseline and week 12). ^b^ *p*-values are produced from repeated measures generalized mixed-effects models adjusted for age, sex, and BMI (group × time interaction).

**Table 6:** Possible or probably related AEs by class and term.

AE Class	Diagnosis or Symptom	Placebo (*n* = 50)	Nutraceutical (*n* = 50)
Gastrointestinal	*n*	0 (0%)	4 (10%)
	Stomach pains	0 (0%)	1 (2%)
	Increased bowel movements	0 (0%)	1 (2%)
	Nausea	0 (0%)	2 (4%)
Neurological	*n*	1 (2%)	2 (4%)
	Low mood	0 (0%)	1 (2%)
	Tiredness	1 (2%)	0 (0%)
	Worsened sleep	0 (0%)	1 (2%)
Dermatological	*n*	0 (0%)	1 (2%)
	Minor skin discoloring (orange)	0 (0%)	1 (2%)
All adverse events	*n*	1 (2%)	4 (10.0%)
Number of participants experiencing treatment-related AE *		1 (2%)	5 (10%)

* Some participants experienced more than one treatment-related AE.

## Data Availability

The raw data collected for this study can be requested by contacting the corresponding author.

## Supplementary Materials

The following supporting information can be downloaded at: https://www.mdpi.com/article/10.3390/nu16111770/s1, Supplementary tables: Table S1. Cognitive assessments conducted and their order of presentation, Table S2. Calculations used for cognitive performance outcomes, Table S3. Scores on Individual Cognitive Tasks (estimated marginal means), Table S4. Change in Safety Bloods from Week 0 to 12.
