# Supplementary material for: An Examination into the Effects of a Nutraceutical Supplement on Cognition, Stress, Eye Health, and Skin Satisfaction in Adults with Self-Reported Cognitive Complaints: A Randomized, Double-Blind, Placebo-Controlled Trial
Source: Nutrients. 2024 Jun 5;16(11):1770. doi: 10.3390/nu16111770 (PMC11174377; doi:10.3390/nu16111770)
Supplement: Supplementary file 1 [file nutrients-16-01770-s001.zip › nutrients-3025316-supplementary.pdf]

Table S1. Cognitive assessments conducted and their order of presentation.

Cognitive task	Description	Scoring
1. RAVLT (6 trials + interference trial)	In the RAVLT, the examiner reads aloud a list of 15 words at the rate of one word per second. The participant is then asked to repeat all words from the list that he/she can remember. This procedure is repeated a total of five times. The examiner then presents a second list of 15 words (interference list), allowing the participant only one attempt to recall this new list. Immediately following this, the participant is asked to remember as many words as possible from the first list.	Number of correctly recalled words for each trial
2. Computerised location learning	A 5 x 5 grid is presented in which 10 of the squares contain pictures of objects. The participant is asked to remember the locations of these objects within the grid. On five occasions, they are then presented with a blank 5 x 5 grid with the objects displayed to the right of the screen and are required to relocate the objects to the correct location.	Displacement score
3. Simple reaction time	On 50 occasions, an upwards pointing arrow is displayed on the screen at irregular intervals. Participants respond as quickly as possible when they see the arrow appear	Reaction time (ms)
4. Digit vigilance	A fixed number appears on the right of the screen and a series of changing numbers appear on the left side of the screen. Participants are required to make a response when the number on the left matches the number on the right.	Accuracy (%) and reaction time for the correct responses (ms)
5. Choice reaction time	Arrows pointing left and right appear on the screen at irregular intervals. The participant is required to indicate the direction of the arrow as quickly as possible whenever an arrow is displayed, by pressing the corresponding button.	Accuracy (%) and reaction time for the correct responses (ms)
6. Numeric working memory	A series of numbers are displayed on the screen, one at a time. Participants are required to memorise these numbers. Once the series is complete, numbers are displayed one at a time and participants are required to indicate if each number was presented in the previous list or not. In this task, three trials are completed, with five target numbers in each trial.	Accuracy (%) and reaction time for the correct responses (ms)
7. Corsi Blocks	Nine blue squares on a black background are displayed on the screen. Some of the blue squares change to red and back to blue again in a sequence. Participants are required to remember this sequence. The task is repeated five times at each level of difficulty with the sequence span increasing from 4 upwards, until the participant can no longer correctly recall the sequences.	Span score
8. Delayed location recognition	A blank 5 x 5 grid is presented with the same objects to the right of the screen as shown in the computerised location learning task. The participant is required to relocate the objects to the position on the grid presented during the computerised locations learning task without seeing them again.	Displacement score
9. RAVLT (delayed recall)	The participant is asked to repeat all the words that he/she can remember from the RAVLT task.	Number of correctly recalled words
10. RAVLT (recognition trial)	The participant is presented with a sheet of paper that contains a list of 50 words. He/she is required to mark the words that were presented in the previous trials.	Number of correctly recognised words

Table S2. Calculations used for cognitive performance outcomes

Cognitive Skills	Tasks used in calculations
Episodic memory (mean percentage of cognitive tasks)	1. Numeric working memory (percentage correct)<br>2. Location learning recall (percentage accuracy) [90 (maximum score) - displacement score/ 90 (maximum score) x 100]<br>3. RAVLT delayed recall (number recalled / 15 x 100)
Working memory (mean percentage of cognitive tasks)	1. Corsi blocks (percentage) [span score/ 15# x 100]<br>2. Numeric working memory (percentage correct)<br># 15 = maximum sequence
Speed of information processing (mean reaction time of cognitive tasks)	1. Simple reaction time (reaction time in milliseconds)<br>2. Choice reaction time (reaction time in milliseconds of correct responses)<br>3. Numeric working memory (reaction time in milliseconds of correct responses)<br>4. Digit vigilance (reaction time in milliseconds of correct responses)
Accuracy of attention (mean percentage of cognitive tasks)	1. Choice reaction time (percentage correct)<br>2. Digit vigilance (percentage correct)
Visuospatial learning	1. Location learning task (displacement score during 5 trials)

Table S3. Scores on Individual Cognitive Tasks (estimated marginal means)

		Placebo (n=44)				Nutraceutical (n=45)				p-value[b]
		Week 0	Week 12	% Change	p-value[a]	Week 0	Week 12	% Change	p-value[a]	
Digit Vigilance (% correct)	Mean	93.50	93.33	-0.18	0.859	94.81	94.54	-0.28	0.772	0.938
	SE	1.57	1.59			1.55	1.57			
Digit Vigilance (reaction time for correct responses in ms)	Mean	473.37	468.74	-0.98	0.179	465.35	468.02	0.57	0.427	0.13
	SE	5.37	5.41			5.14	5.27			
Choice reaction time (% correct)	Mean	97.24	96.94	-0.31	0.372	96.95	97.31	0.37	0.283	0.165
	SE	0.45	0.46			0.44	0.45			
Choice reaction time (reaction time for correct responses in ms)	Mean	522.76	509.19	-2.60	0.144	515.67	507.18	-1.65	0.35	0.701
	SE	16.34	16.16			15.70	15.68			
Numeric Working Memory (% correct)	Mean	92.87	93.15	0.30	0.755	92.23	95.60	3.65	< .001	0.014
	SE	0.96	0.99			0.93	0.99			
Numeric Working Memory (reaction time for correct responses in ms)	Mean	993.27	919.70	-7.41	0.002	1006.65	980.92	-2.56	0.295	0.147
	SE	34.00	32.22			33.61	33.49			
Location learning (total of displacement scores from trials 1 to 5)	Mean	43.94	26.93	-38.71	< .001	45.36	29.11	-35.83	< .001	0.715
	SE	5.01	3.15			5.05	3.32			
Location learning recall (displacement score)	Mean	2.84	2.19	-23.03	0.148	4.37	1.09	-75.04	< .001	< .001
	SE	0.66	0.58			0.84	0.37			
Corsi Blocks (span score)	Mean	5.67	5.78	1.94	0.456	5.69	5.56	-2.28	0.347	0.234
	SE	0.16	0.16			0.15	0.15			
Simple reaction time (ms)	Mean	312.51	305.24	-2.33	0.172	309.48	300.10	-3.03	0.072	0.765
	SE	8.47	8.43			8.18	8.07			
RAVLT recognition score	Mean	11.64	12.06	3.65	0.269	10.68	11.09	3.79	0.245	0.976
	SE	0.45	0.48			0.41	0.43			
RAVLT delayed recall	Mean	10.55	12.23	15.92	< .001	9.54	11.14	16.75	< .001	0.857
	SE	0.44	0.52			0.39	0.46			

Results (estimated means) are generated from generalised mixed-effects models adjusted for age, sex, and BMI. [a]P-values are generated from repeated measures generalised mixed-effects models adjusted for age, sex, BMI (time effects baseline and week 8). [b]P-values are generated from repeated measures generalised mixed-effects models adjusted for age, sex, and BMI (time x group interaction).

Table S4. Change in Safety Bloods from Week 0 to 12

		N	Mean	SE	p-value^
**Liver function**					
Aspartate transaminase (AST)	Placebo	41	-2.76	2.40	0.243
	Nutraceutical	44	0.20	0.96	
Alanine transaminase (ALT)	Placebo	41	-1.07	1.21	0.500
	Nutraceutical	44	0.16	1.34	
Alkaline phosphatase (ALP)	Placebo	41	-2.90	1.25	0.935
	Nutraceutical	44	-3.11	2.20	
Gamma-Glutamyl Transferase (GGT)	Placebo	41	0.73	1.14	0.436
	Nutraceutical	44	-2.32	3.61	
Bilirubin	Placebo	41	0.56	0.49	0.540
	Nutraceutical	44	0.16	0.43	
Total protein	Placebo	41	0.12	0.54	0.862
	Nutraceutical	44	0.25	0.50	
Globulin	Placebo	41	-0.27	0.45	0.994
	Nutraceutical	44	-0.27	0.36	
Albumin	Placebo	41	0.39	0.37	0.789
	Nutraceutical	44	0.52	0.32	
**Renal Function**					
Urea	Placebo	41	0.20	0.24	0.967
	Nutraceutical	44	0.18	0.22	
Creatinine	Placebo	41	1.37	1.34	0.625
	Nutraceutical	44	0.43	1.36	
Estimated Glomerular Filtration Rate (eGFR)	Placebo	41	-1.66	1.21	0.527
	Nutraceutical	44	-0.70	0.91	
Sodium	Placebo	41	-0.32	0.36	0.421
	Nutraceutical	44	0.09	0.35	
Potassium	Placebo	41	-0.07	0.06	0.427
	Nutraceutical	44	0.00	0.07	
Chloride	Placebo	41	0.90	0.37	0.989
	Nutraceutical	44	0.91	0.33	
Bicarbonate	Placebo	41	-0.61	0.39	0.036
	Nutraceutical	44	0.41	0.27	
Anion gap	Placebo	41	-0.71	0.51	0.439
	Nutraceutical	44	-1.18	0.35	

^ Independent samples T-test
